# Socioeconomic disparity in prefrontal development during early childhood

**DOI:** 10.1038/s41598-019-39255-6

**Published:** 2019-02-22

**Authors:** Yusuke Moriguchi, Ikuko Shinohara

**Affiliations:** 1 Graduate School of Education, Kyoto University, Yoshidahoncho, Kyoto, 606-8501 Japan; 2 Japan Science and Technology Agency, PRESTO/Sakigake, Honcho4-1-8, Kawaguchi, Saitama, 332-0012 Japan; 3 Department of Education, Joetsu University of Education, Yamayashikicho, Joetsu, 943-8501 Japan; 4 National Institute for Educational Policy Research of Japan, Kasumigaseki 4, Chiyodku, Tokyo, 100-8959 Japan

## Abstract

Socioeconomic status (SES) has a powerful influence on cognitive, social and brain development. Children from low-SES backgrounds show poor executive function (EF). However, it is unclear if there is a SES-dependent disparity in functional brain development. The present study examined whether the SES of preschool children (N = 93) is associated with prefrontal activation during cognitive shifting tasks as measured by near-infrared spectroscopy. Low-SES children did not show activation in lateral prefrontal regions during the tasks, whereas middle- and high-SES children showed prefrontal activations, although no differences were found in terms of behavioural performance. These results suggest that SES can affect the functional development of the prefrontal regions. In this study, we discuss the practical implications of the results.

## Introduction

Socioeconomic status (SES) is an important factor influencing cognitive, social and brain development. Recent studies have consistently shown poor performance on executive function (EF) tasks by low-SES children compared with that by middle- and high-SES children during childhood and adolescence^1–5^. EF comprises several cognitive domains such as inhibition, cognitive shifting and working memory which facilitate goal-directed control of thoughts and actions^6–8^. Low EF during early childhood is a major risk factor for poor academic and social function in later life^9–13^.

Several models have been proposed to explain the relationship between SES and cognitive development^14^. Research on EF generally supports the roles of parenting quality, home environment and children’s stress experiences in mediating the relationship between SES and EF^15–17^. Theoretically, poverty could directly disrupt brain development, leading to impaired self-regulation and EF^18^. In animal models, adverse environmental conditions, including chronic stress and lack of stimulation, impair the structural and functional development of the prefrontal cortex^19,20^. An anatomical evidence reports that the human prefrontal cortex may be affected by childhood poverty^21–23^. Moreover, fMRI studies found that lower-SES, school-aged children and adolescents showed different patterns of prefrontal activation than higher-SES children in tasks which required executive control^5,24,25^. Nevertheless, it is still unknown whether SES also influences functional brain development during early childhood. Specifically, despite dramatic changes in EF during preschool years; the strong relationship between EF and functional development of the lateral prefrontal cortex in young children and the fact that EF during preschool years predicts later academic achievement and peer relationships^11–13,26–28^, few functional studies have compared prefrontal activity among young children of different SES.

Recently, electrophysiological studies have shown some association between SES and potential event-related components which reflect EF (e.g. N2) in preschool children^29,30^. However, event-related potential (ERP) has limited spatial resolution and provides an indirect index of the prefrontal development; therefore, whether the prefrontal activations are affected by childhood poverty is unclear. To address this important issue, we examined the prefrontal activation patterns of 93 preschool children of varying SES during a cognitive shifting task using near-infrared spectroscopy (NIRS) and evaluated the statistical relationships among task performance, prefrontal activation and SES.

We used a cognitive shifting task primarily for three reasons. First, several previous studies have reported that children develop cognitive shifting during early childhood and that the prefrontal regions are activated during cognitive shifting tasks^6,31–33^. Second, a meta-analysis from behavioural studies reported that the average correlation (effect sizes) between cognitive shifting and SES in children was comparable to that between working memory and SES; further, it was higher than the correlation between inhibition and SES^34^. Thus, although few studies examined the effects of SES on cognitive shifting^2^, we hypothesised that SES would affect prefrontal activations in young children.

In addition, previous studies have shown that parenting mediates the relationship between SES and EF^16^, so we assessed parenting style as a possible mediator. We predicted that the children from low-SES families would show weaker prefrontal activation compared with those from higher-SES families. Moreover, considering the mounting evidence that brain measures are more sensitive to SES than behavioural measures^14^, we predicted that prefrontal activation would be more sensitive to SES than EF task performance.

## Methods

### Participants

Participants were recruited from a nursery school of a small city of Osaka Prefecture. Of these, four children failed to complete the experiment and the parents of three participants disagreed to report their SES. Finally, a total of 93 preschool children (45 males and 48 females) participated in this study (mean age = 59.8 months, SD = 10.5, range = 42–77 months). These Japanese-speaking children had no known developmental abnormalities. Informed consent was obtained from their parents prior to their involvement in the study, which was conducted in accordance with the principles of the Declaration of Helsinki and was approved by the Ethics Review Board of Joetsu University of Education (2015–1).

### Materials

#### SES

SES was assessed by maternal education and family income. Parents’ education level was assigned a value from 1 to 5 as follows: 1, less than high school; 2, high school; 3, some college; 4, undergraduate degree and 5, graduate level. Second, parents reported a self-reported measure of family income in 12 categories considering their reluctance in reporting the exact income (in Japanese yen): 0–1,000,000; 1,000,001–2,000,000; 2,000,001–3,000,000; 3,000,001–4,000,000; 4,000,001–5,000,000; 5,000,001–6,000,000; 6,000,001–7,000,000; 7,000,001–8,000,000; 8,000,001–9,000,000; 9,000,001–10,000,000; 10,000,001–15,000,000 and >15,000,001. The income was assigned a value of 1–12 and adjusted by household size.

We created two measures to categorise SES based on previous studies^2^. First, the mother’s educational level and family income were converted to z-scores separately, and then averaged to create the total SES score. Second, considering our aim to clarify whether children of poverty show different behavioural and neurological responses than children belonging to higher-SES families, we defined poverty according to the Organization for Economic Co-operation and Development (OECD) criterion. OECD defines poverty as half the median household income of the total population. In Japan, families with income <¥1,220,000 were defined as poverty. According to the criterion, the proportion of child poverty is approximately 15% in Japan. Given the criterion, we regarded the families to be experiencing poverty if their income was less than the adjusted income of \1,220,000. Fourteen children (8 males) were categorised into a poverty group (mean age = 62.6 months) and 79 children (37 males) into a no-poverty group (mean age = 59.3 months).

#### Parenting style

Reportedly, parenting may mediate the relationship between SES and EF. We used a parenting style questionnaire based on Baumrind^35,36^. This questionnaire assessed two factors: Responsiveness (e.g. expresses affection by hugging and kissing) and Control (e.g. commanding the child). A 4-point Likert-type scale was provided for each item, ranging from never to always. The items used were based on the Parenting Styles and Dimensions Questionnaire^35^. The Japanese questionnaire is a shorter version including 15 items that loaded the Responsiveness and Control factors. This version has been previously validated^37^. Further, we calculated McDonald’s omega (ω) to estimate reliability for the Responsiveness and Control scores^38^ and found both scores to be reliable (Responsiveness ω = 0.65, Control = 0.69).

#### Behavioural test of EF

**Figure 1:**
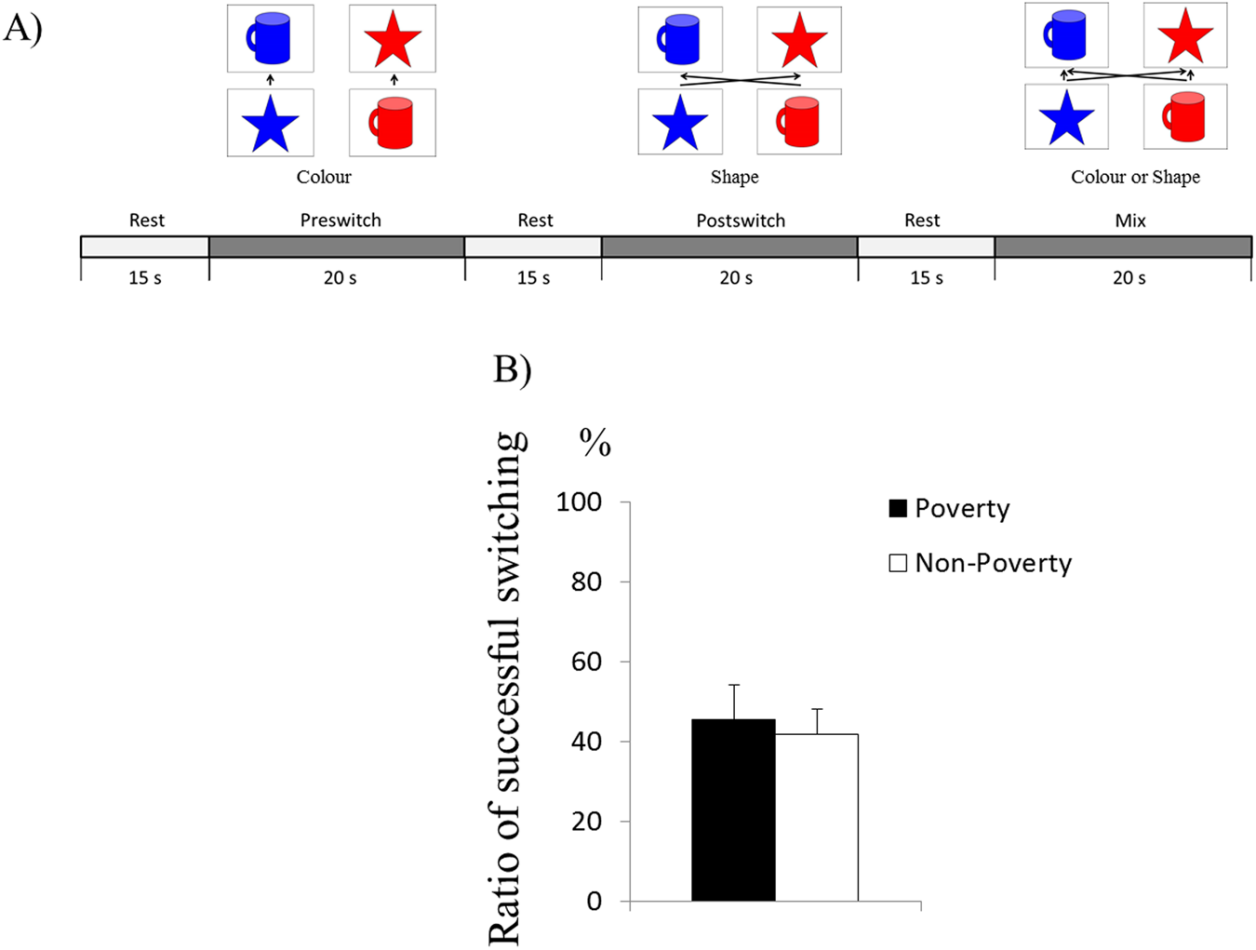
Experimental design. (**A**) Experimental sequence for the Dimensional Change Card Sorting task. Children were instructed to match cards according to colour or shape. (**B**) Behavioural results show no difference in terms of switching accuracy during the five transitions in task instruction (sort by colour to sort by shape or vice versa).

We used a modified version of the Dimensional Change Card Sort (DCCS) task from the NIH toolbox adapted for NIRS measurements to assess the effects of SES on EF^33,39^ (Fig. 1A). This task included target and test cards. The target cards matched the test cards in one dimension such as the shape depicted but not in the second dimension such as colour, and the rule for matching was changed according to the session or experimenter’s instruction. For instance, a target card depicting a red star could match a blue star (shape) or red cup (colour). The experiments included three different pairs of target and test cards.

The children performed three consecutive test sessions. Each session comprised rest (15 s), pre-switch (20 s), second rest (15 s), post-switch (20 s), third rest (15 s) and mix (20 s) phases. During the rest phase, children were asked to be still. Although some previous studies used control phases with simple tasks instead of rest phases, control and rest phases showed similar results in terms of the prefrontal activations during DCCS tasks^27,39^. During the pre-switch phase, the children were asked to sort the test cards according to the first rule (e.g. colour). During the post-switch phase, they were asked to sort the cards according to the second rule (e.g. shape). Lastly, during the mix phase, the children were asked to sort cards according to the instructed rule (colour or shape). In each phase, the children were given the rule before each trial (e.g. colour). The rule order (e.g. colour first) during the pre-switch and post-switch phases was held constant across the three sessions for each child, but the rule order was counterbalanced across children. The rule order during the mix phase was fixed: POST (rule for the post-switch phase), POST, PRE (rule for the pre-switch phase), POST, POST, PRE, POST and POST.

The dependent measure was the percentage of successful switches, which was calculated as a measure of total performance because the pre-switch and post-switch trials are generally considered easy for older children. The passing criterion was 90% correct in the pre-switch and post-switch phases. The children had to switch the rule once between the pre-switch and post-switch phases and four times during the mix phase. Thus, the number of successful switches was calculated out of five.

### NIRS recordings

A multichannel NIRS unit (OEG-16; Spectratech Inc., Tokyo, Japan) operating at wavelengths of 770 and 840 nm was used to measure temporal changes in the concentrations of oxygenated haemoglobin (oxy-Hb) and deoxygenated haemoglobin (deoxy-Hb) during the DCCS tasks. The NIRS probes included 12 optodes constituting 16 channels. The probes were placed on the lateral prefrontal areas of each hemisphere. Each channel comprised one emitter optode and one detector optode located 3 cm apart. The temporal resolution at each channel was approximately 666 ms.

Regions of interest near the electrode positions F3 and F4 of the International 10–20 system were predetermined based on previous studies showing activation of these areas during DCCS tasks^40,41^. The spatial resolution of NIRS is relatively low, so channels 2, 4 and 5 were defined as corresponding to the right lateral prefrontal region and channels 11, 13 and 14 as corresponding to the left lateral prefrontal region. For technical reasons, channel 11 did not function well, and we failed to collect data from 41 participants in this channel. Thus, this channel was excluded from the analysis. We successfully collected the data of all participants in the other channels.

We measured changes in oxy-Hb and deoxy-Hb in the lateral prefrontal areas during the rest phases and each of the task phases. In terms of the data analyses, first, the data were filtered with moving average (data points: 5) and baseline correction was performed using linear fitting. Further, the NIRS signal was separated into functional (i.e. brain activation) and systematic (i.e. physiological noise) components based on a negative or positive linear relationship between oxy-Hb and deoxy-Hb changes^42^. Lastly, average changes in oxy-Hb and deoxy-Hb during the rest and task phases were calculated for each channel in each subject.

### Data

The datasets supporting this article have been uploaded as part of the Supplementary Material.

### Statistical analysis

All statistical analyses were performed using R statistical software (Version 3.4.1, R Core Team, 2017). First, we analysed the relationship between SES and the behavioural measures of EF. Specifically, we separately analysed the total SES scores and poverty. The behavioural measures of EF and parenting measures were not normally distributed; thus, we conducted Spearman’s correlational analyses for total SES scores and poverty. When we found significant correlation among variables, we conducted further analyses to assess whether and how SES affected children’s EF.

We analysed the relationships among SES, parenting and prefrontal activations in each channel. We conducted preliminary correlational analyses to examine relationships among variables and found that parenting measures and age (months) were not significantly correlated with the measures of the prefrontal activations (*ps* > 0.162). Moreover, behavioural measures of EF were not significantly correlated with prefrontal activations (*ps* > 0.189). We did not consider the variables in further analyses.

We directly analysed the relationship between SES and prefrontal activations. First, we examined whether there was a liner relationship between the total SES scores and the prefrontal activations in the prefrontal regions. We used the difference scores between the prefrontal activations during the aggregate task phase and the prefrontal activations during the aggregate rest phase as the indices of task-related prefrontal activations. Spearman’s correlational analysis was used to assess the relationship between the total SES scores and difference scores.

Second, we analysed whether the prefrontal activations in the prefrontal regions differed across the poverty and no-poverty groups. Change in oxy-Hb (Δoxy-Hb) was analysed using three-way mixed ANOVA with phases (rest vs. task) and channels (channels 2, 4, 5, 13 and 14) as the within-subject factors and poverty (poverty vs. no-poverty group) as the between-subject factor. Post-hoc analyses using Bonferroni method was performed for variables showing significant interaction.

## Results

### Effect of SES on behavioural tests of EF

Descriptive results are summarised in Table 1. First, we examined the relationship between total SES score, parenting and the proportion of successful switches. Our correlational analyses revealed that the total SES score was significantly correlated neither with the parenting style (Responsiveness and Control, Spearman’s rho, *r* = 0.192 and 0.095, *p*s > 0.065) nor with the proportion of successful switches (Spearman’s rho, *r* = 0.091, *p = *0.384). We did not further analyse the effect of total SES score on EF.

**Table 1:** Descriptive statistics of each variable (Mean (SD)).

Variable	Total	No-Poverty/Poverty
**SES**		
Maternal education (1–5)	2.62 (0.90)	2.66 (0.90)/2.43 (0.85)
Income (adjusted by household size)	2.66 (1.33)	2.97 (1.19)/0.89 (0.24)
Total SES (z transformation)	0 (0.799)	0.137 (0.763)/−0.775 (0.505)
Parenting style		
Responsiveness (1–4)	3.30 (0.30)	3.32 (0.31)/3.17 (0.27)
Control (1–4)	2.23 (0.37)	2.27 (0.38)/2.06 (0.27)
DCCS (0–100)	42.49 (31.73)	41.93 (31.88)/45.63 (31.91)

Note. Total SES is the average score of the z score of mother’s educational level and the z score of family income. DCCS represents dimensional change card sort.

Next, we examined whether poverty was correlated with parenting style and EF. The results revealed that poverty was significantly correlated with Control (Spearman’s rho, *r* = 0.217, *p* = 0.037) but not with Responsiveness (Spearman’s rho, *r* = 0.183, *p* = 0.080) and the proportion of successful switches (Spearman’s rho, *r* = −0.064, *p* = 0.539) (Fig. 1B). Moreover, Control (Spearman’s rho, *r* = 0.316, *p* = 0.002), but not Responsiveness (Spearman’s rho, *r* = 0.015, *p* = 0.887), was significantly correlated with the proportion of successful switches. Age (months) was significantly correlated with the proportion of successful switches (Spearman’s rho, *r* = 0.668, *p = *0.001); therefore, we conducted a partial correlational analyses about the relationship between Control and the proportion of successful switches after controlling for age (months). We found a significant correlation between Control and the proportion of successful switches after controlling for age (months; Spearman’s rho, *r* = 0.348, *p* = 0.001).

**Figure 2:**
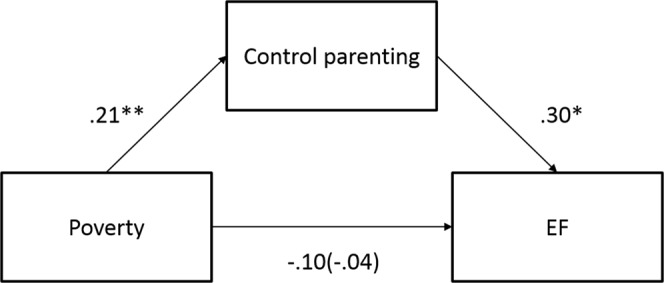
A mediation model.

Although there was no direct association between SES and the percentage of successful switches, poverty was significantly correlated with parenting styles with the parents in poor families tending to show lower Responsiveness and Control. Thus, we conducted a mediation analysis to assess whether poverty indirectly affected successful switching through parenting style using a Sobel test (Fig. 2). A mediation model revealed no significant effect of Control parenting as a mediator (ab = 0.062, z = 1.73, *p* = 0.085).

### NIRS results

We analysed the relationships between SES and prefrontal activation in each channel. Correlational analyses revealed that the total SES score was not significantly correlated with the difference scores in each channel (Spearman’s rho *rs* < 0.091, *p* > 0.388).

**Figure 3:**
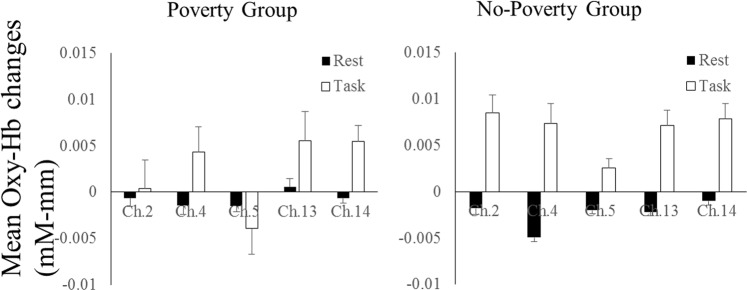
Mean oxy-Hb changes within the right (channels 2, 4 and 5) and left (channels 13 and 14) lateral prefrontal areas in the poverty and no-poverty groups during the rest and task phases of the DCCS tasks. Error bars indicate standard error.

**Figure 4:**
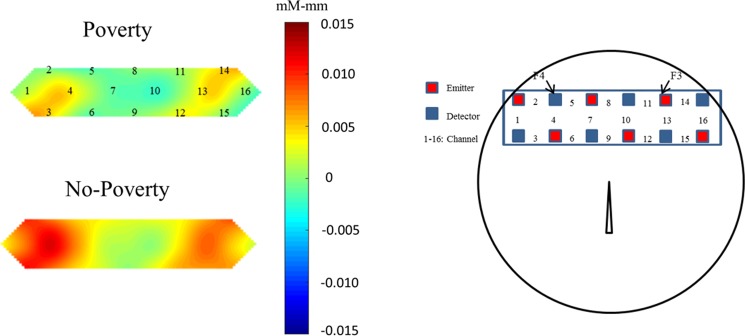
Distinct neural activation patterns in lateral prefrontal regions of preschool children from the poverty and no-poverty groups. Averaged overall near-infrared spectroscopy data were compared between task and rest phases. Each channel consisted of one emitter optode and one detector optode. The regions of interest were located near F3 and F4 of the 10/20 system, corresponding to channels 2 4 and 5 (right hemisphere) and channels 13 and 14 (left hemisphere), respectively. The numbers (1–16) indicate the channels of the NIRS probe. Low-SES children exhibited hypoactivation in the lateral prefrontal region.

Then, we analysed the effect of poverty (Fig. 3). The results of mixed ANOVA revealed significant interaction between phases and channels [*F* (4, 91) = 3.269, *p* = 0.012, *η*^2^ = 0.035] and between poverty and phase [*F* (1, 91) = 3.967, *p* = 0.049, *η*^2^* = *0.042]. Post-hoc analyses for the interaction between phases and channels revealed that children exhibited significant oxy-Hb changes during the task phases than during the rest phases in the right (channels 2 and 5) and left (channels 13 and 14) prefrontal regions (*ps* < 0.020). More importantly, post-hoc analyses for the interaction between poverty and phases revealed that children in the no-poverty group exhibited significant oxy-Hb changes during the task phases than during the rest phases (*p* = 0.001), whereas those in the poverty group showed no significant oxy-Hb changes in between the rest and task phases (*p* = 0.108) (Figs 3 and 4).

## Discussion

Research involving animal models and older children have revealed that environmental deprivation or poverty can impair functional development of the prefrontal cortex^20,24^. Structural MRI studies in young children have shown that cortical thickness in prefrontal regions and macro- and microstructural properties of white matter tracts implicated in EF differ depending on SES^23,43^. Moreover, young lower-SES children exhibited undeveloped event-related potential patterns compared with higher-SES children during tasks involving attention and inhibition^29,30^. However, it was unclear whether and how poverty affects functional development of the prefrontal cortex in young children. The present study provides the evidence for a relationship among EF deficits, prefrontal hypoactivation and low SES in young children.

Preschool children from low-SES families showed no significant activation in the prefrontal regions during cognitive shifting tasks compared with the children from middle- and higher-SES families. At the behavioural level, however, SES did not affect performance, in contrast to the results reported by previous studies^1,3^. This may be due to that the tasks used in this study may be relatively less sensitive to SES differences. The average correlation between cognitive shifting and SES in children has been found to be moderate^34^, but a previous study reported the correlation between DCCS tasks and SES to be specifically small (*r* = 0.17)^44^.

Nevertheless, SES was directly related to the functional development of the prefrontal cortex as evidenced by neural activity. We used two measures of SES, total SES score as a continuous measure and poverty/no-poverty as a categorical measure, but found a significant relationship with prefrontal cortex activation only for poverty. The children from low-income families showed no significant activations in the prefrontal region compared with those from middle- and high-income families. Perhaps, children from low-income families involve other brain regions, such as the parietal regions, to perform the tasks. Although the effects of SES on health and brain development show a gradient, the effect is steeper at the low end of SES^45,46^. Thus, our results are consistent with those of previous studies.

Our results support the view that brain measures are more sensitive to poverty than behavioural measures^2^. Adequate childcare can support and improve children’s self-control at the behavioural level. However, an SES disparity may still be evident in brain function, consistent with the theory that poverty impedes the development of self-regulation through the effects of chronic stress on the underlying brain circuits^18^. Thus, neuroimaging measures may reveal the adverse influence of poverty on neural development in the absence of obvious behavioural manifestations in childhood. Nonetheless, such developmental abnormalities may exert lasting influences on cognition, academic achievement and life outcome.

A previous study reported a higher prefrontal activation in low-SES school-aged children than in middle- and high-SES children in a novel stimulus-response learning^24^, which is inconsistent with our results. A possible reason for this outcome is that efficiency in some EF tasks is associated with prefrontal activation increases, whereas that of other tasks is associated with prefrontal activation decreases. In terms of DCCS tasks, better performance was associated with increased prefrontal activity during DCCS tasks^27,39,41^, whereas in the stimulus-response learning tasks, better performance was associated with decreased prefrontal activity. Moreover, preschool age is considered a period in which children begin engaging the prefrontal regions during EF tasks. Younger children who present difficulty while performing the tasks do not exhibit prefrontal activations, and these activations become stronger with growth^27,31^. Given the facts, we suggest that weaker activations in low-SES children are linked to developmental delay.

We did not find the significant correlations between EF and the prefrontal activations, and between age in month and the prefrontal activations. This was inconsistent with the previous evidence that behavioural performances of DCCS tasks were correlated with the prefrontal activations, and older children showed the stronger activations in the prefrontal regions compared to younger children during the tasks^27,31^. One difference between studies is the task structure. The previous studies included the preswitch and postswitch phases, and the performances during the postswitch phases were correlated with the prefrontal activations^27^. On the other hand, the present study included the preswitch, postswitch, and mix phases, and calculated the aggregated scores of the phases. Such differences may lead to the different results across studies. Moreover, the relationship between EF performances and the prefrontal activations is sometimes non-linear. Higher performances may not be related to stronger activations in the prefrontal regions^47^. This may be true for the relationship between age and the prefrontal activations.

Finally, our results could contribute to the development of improved support programmes for children from low-SES families. Several programmes are available to enhance and support self-regulation and EF in children from low-SES families^48^. However, most of these programmes only assess behaviour during a relatively brief developmental window, while effects on the underlying neuronal functions may be sustained. Our results clearly show that an SES disparity can still be detected by neural activity measures in the absence of behavioural differences. Thus, it is possible that a programme enhances EF at the behavioural level, but not the neural level, in part because of the limited range of cognitive tests suitable for very young children. We propose that researchers should instead consider neural measures to monitor programme outcomes in test populations, particularly low-SES children.

## Supplementary information

Dataset 1
